# Notch1 deficiency decreases hepatic lipid accumulation by induction of fatty acid oxidation

**DOI:** 10.1038/srep19377

**Published:** 2016-01-20

**Authors:** No-Joon Song, Ui Jeong Yun, Sunghee Yang, Chunyan Wu, Cho-Rong Seo, A-Ryeong Gwon, Sang-Ha Baik, Yuri Choi, Bo Youn Choi, Gahee Bahn, Suji Kim, So-Mi Kwon, Jin Su Park, Seung Hyun Baek, Tae Joo Park, Keejung Yoon, Byung-Joon Kim, Mark P. Mattson, Sung-Joon Lee, Dong-Gyu Jo, Kye Won Park

**Affiliations:** 1 Department of Food Science and Biotechnology, Sungkyunkwan University, Korea; 2 School of Pharmacy, Sungkyunkwan University, Korea; 3 Department of Biotechnology, Graduate School of Life Sciences & Biotechnology, BK21-PLUS program, Korea University, 136-713 Seoul Korea; 4 Department of Internal Medicine, Graduate School of Medicine, Gachon University of Medicine and Science; 5 School of Nano-Bioscience and Chemical Engineering, Ulsan National Institute of Science and Technology; 6 Department of Genetic Engineering, Sungkyunkwan University, Korea; 7 Laboratory of Neurosciences, National Institute on Aging Intramural Research Program, Baltimore, Maryland, USA

## Abstract

Notch signaling pathways modulate various cellular processes, including cell proliferation, differentiation, adhesion, and communication. Recent studies have demonstrated that Notch1 signaling also regulates hepatic glucose production and lipid synthesis. However, the effect of Notch1 signaling on hepatic lipid oxidation has not yet been directly investigated. To define the function of Notch1 signaling in hepatic lipid metabolism, wild type mice and Notch1 deficient antisense transgenic (NAS) mice were fed a high-fat diet. High-fat diet -fed NAS mice exhibited a marked reduction in hepatic triacylglycerol accumulation compared with wild type obese mice. The improved fatty liver was associated with an increased expression of hepatic genes involved in fatty acid oxidation. However, lipogenic genes were not differentially expressed in the NAS liver, suggesting lipolytic-specific regulatory effects by Notch1 signaling. Expression of fatty acid oxidative genes and the rate of fatty acid oxidation were also increased by inhibition of Notch1 signaling in HepG2 cells. In addition, similar regulatory effects on lipid accumulation were observed in adipocytes. Taken together, these data show that inhibition of Notch1 signaling can regulate the expression of fatty acid oxidation genes and may provide therapeutic strategies in obesity-induced hepatic steatosis.

The worldwide increase in obesity is the leading cause of metabolic syndrome^1^,^2^. Excess accumulation of triacylglycerols and cholesterol in obesity can impede insulin signaling and cause cardiovascular diseases. Although active investigation into the etiology of and therapeutic interventions for obesity are in progress, better therapeutic strategies for obesity and its related metabolic syndromes still remain to be elucidated^1^,^3^,^4^.

Adipogenesis is determined by a cascade of transcriptional regulators and a number of signaling pathways including Wnt, Hedgehog, BMP, TGF-β, and Notch^5^,^6^,^7^. Activation of the Wnt, Hedgehog, and TGF-β signaling pathways have been shown to stimulate adipogenesis and adipocyte differentiation^8^. Activation of BMP signaling can promote adipocyte differentiation^8^. Activation of these pathways is relatively clear, however, the role of Notch signaling in the control of adipogenesis remains unclear.

Notch signaling is a conserved pathway that regulates cell proliferation, differentiation, self renewal potential, apoptosis, inflammatory response, and cell-fate decision^9^,^10^,^11^,^12^. Notch signaling pathways are complex and involve four Notch receptors and five ligands of the Jagged/Delta-like families. Activated Notch receptors by cognate interaction with ligands can lead to cleavage, release, and nuclear entry of the Notch intracellular domain (NICD). In the nucleus, activated NICD induces transcription of target genes such as Hairy enhancer of split (HES) and the Hes-related (Hey) family^13^. Recent studies have shown that Notch1 signaling also regulates hepatic glucose and lipid accumulation. Notch1 haploinsufficiency can increase insulin sensitivity by suppressing glucose 6-phosphatase (G6p) expression in the liver^14^. Forced expression of NICD1 in the liver increases fatty acid synthase (Fas) expression and induces hepatosteatosis, whereas NICD1 increases intestinal lipid accumulation by inducing RPL29 and Abhd1 expression^14^,^15^. In addition, recent studies have also shown that inhibition of Notch1 signaling in the liver decreases mTorc1 stability, resulting in decreased lipogenesis and protection from diet-induced fatty liver^13^. These results indicate that Notch1 plays a significant role in glucose and lipid metabolism.

In the present study, Notch1-deficient Notch1 antisense (NAS) mice were fed a high-fat diet to determine the effect of Notch1 signaling on hepatic lipid accumulation and expression of lipogenic and oxidative genes. Our data show that inhibition of Notch1 signaling suppresses diet induced hepatic lipid accumulation, likely through increased fatty acid oxidation. These data further suggest the diverse but conserved biological actions of Notch1 signaling in metabolism.

## Results

### Notch1 expression is increased in obese and diabetic fatty livers

**Figure 1:**
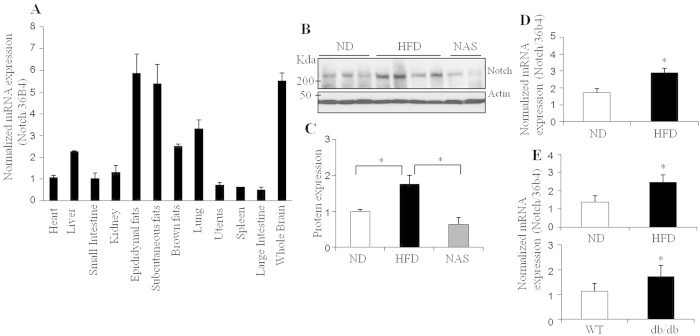
A high-fat diet increases Notch1 expression in liver. (**A**) Tissue distribution of *Notch1* mRNA expression in mice; (**b,c**) Hepatic Notch1 protein expression (**B**) and quantification (**C**) in normal diet-fed (ND), high-fat diet-fed mice (HFD), and high-fat diet-fed Notch1 antisense transgenic (NAS) mice were detected by Western blotting; (**D**) Hepatic *Notch1* mRNA expression was measured by real-time PCR in ND- and HFD-fed mice; (**E**) *Notch1* mRNA expression in epididymal fat tissues was measured in HFD obese (upper) and diabetic db/db mice (lower). Data shown represent the mean ± SEM (n = 3 or 4 per group). Statistically significant differences in protein expression between the control ND-fed C57BL/6 and HFD-fed mice, or between non-diabetic C57BL/6 mice (WT) and diabetic (db/db) mice were determined by a Student’s *t*-test (*p < 0.05).

To examine the tissue expression pattern of *Notch1* in mice, real-time PCR was performed. *Notch1* expression was highest in the brain, with surprisingly similar expression levels in adipose tissues, and modest expression in the lung and liver (Fig. 1A). Other tissues also expressed *Notch1*, although their levels were lower than those of white adipose tissue and brain. Consistent with previous data^14^,^16^, the strong expression profiles of Notch1 in fat tissues and liver indicate that Notch1 may play a significant role in glucose and lipid metabolism. To investigate the potential roles of Notch1 in metabolism, Notch1 expression was measured in high-fat diet-fed obese and diabetic mice. Notch1 protein and mRNA expression were greatly elevated in the livers of obese mice (Fig. 1B–D). A similar expression pattern was detected in the white adipose tissue of obese and diabetic mice (Fig. 1E), suggesting a positive correlation between the expression of Notch1 and lipid accumulation and metabolic diseases.

### Inhibition of Notch1 signaling prevents insulin resistance in diet-induced obese mice

**Figure 2:**
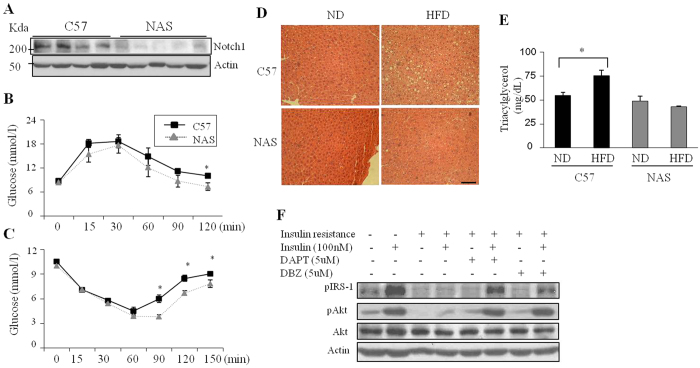
Genetic and pharmacological reduction of Notch1 signaling improves insulin sensitivity and reduces lipid accumulation in liver cells exposed to obesity-promoting fats. (**A**) Reduced hepatic Notch1 expression in NAS mice. Notch1 protein expression from the livers of HFD-fed C57BL/6 (C57) and NAS mice was measured by Western blotting. Glucose tolerance (**B**) and insulin tolerance tests (**C**) were performed in 16-hour-fasted C57BL/6 and NAS mice. The mice analyzed were 21-weeks-old, 12 weeks HFD-fed C57BL/6 and NAS mice (n = 7 of each group); (**D**) H&E stain of liver sections from C57BL/6 and NAS mice; (**E**) Serum triacylglycerol levels from C57BL/6 (black) and NAS mice (gray) were determined; (**F**) Western blotting analysis of insulin signaling proteins from HepG2 cells. An insulin-resistant state was induced in HepG2 cells by treatment with oleic acid and the Notch signaling inhibitors, DAPT and DBZ. Statistically significant differences in NAS mice were determined relative to the control mice by a Student’s *t*-test (*p < 0.05).

Earlier studies have shown that Notch1 inhibition can improve insulin sensitivity^13^,^14^. Therefore, the effects of Notch inhibition in diet-induced obese mice were investigated in the present study. Mice overexpressing Notch1 antisense (NAS) under the control of a mouse mammary tumor virus long terminal repeat promoter were generated and backcrossed to C57BL/6 mice and characterized as described previously^17^. NAS mice with defects in Notch1 expression and downstream signaling have been previously verified by several groups^12^,^18^. NAS and wild type C57BL/6 mice (C57) were fed a high-fat diet (HFD) and a normal chow control diet (ND) for 12 weeks. The inhibition of Notch1 expression in the liver of NAS mice was confirmed by Western blotting (Fig. 2A). Body weight was not consistently different between the NAS and control mice (Fig. S1A). Glucose tolerance (GTT) and insulin tolerance tests (ITT) confirmed that the insulin sensitivity was significantly (*p* < 0.05) improved in NAS mice (Fig. 2B,C). Histological analysis of livers showed less lipid accumulation in the HFD-fed NAS mice compared with the control mice (Fig. 2D). Lower serum triacylglycerol levels in NAS mice fed a HFD further suggest that Notch1 inhibition prevents hepatic steatosis (Fig. 2E). Consistently, elevated levels of free fatty acids in the high-fat diet-fed control mice were significantly (*p* < 0.05) reduced in NAS mice (Fig. S2). Plasma insulin levels and food intake were not significantly (*p* > 0.05) different between the two groups. However, the plasma glucagon level was significantly (*p* < 0.05) decreased in NAS mice (Fig. S1).

To further verify the effect of Notch1 inhibition on insulin sensitivity, HepG2 cells were induced to an insulin-resistant state by treatment with oleic acid, and subsequently treated with the inhibitor of γ-secretase (an enzyme that activates Notch signaling) N-[N-(3.5-difluorophenacetyl)-L-alanyl]-S-phenylglycine t-butylester (DAPT) or dibenzazepine (DBZ). Inhibitory effects of DAPT and DBZ on Notch1 signaling were confirmed in HepG2 cells (Fig. S3). Insulin treatment under resistant conditions resulted in significantly (*p* < 0.05) lower levels of phosphorylated AKT1 and IRS-1 compared with the levels of phosphorylated AKT1 and IRS-1 under normal conditions. Treatment with these inhibitors increased AKT and IRS-1 phosphorylation levels similar to those seen in insulin sensitive states, even under insulin-resistant conditions (Fig. 2F).

### Inhibition of Notch1 signaling induces hepatic oxidative genes in diet-induced fatty liver

**Figure 3:**
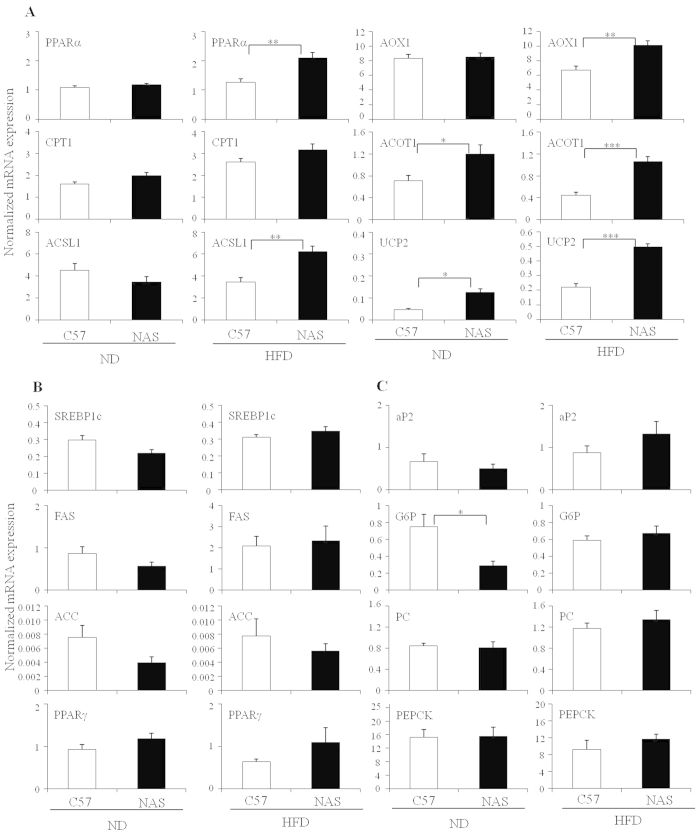
Mice with reduced Notch1 levels exhibit increased expression of genes encoding lipid oxidation proteins. (**A–C**) Hepatic expression of oxidative, lipogenic, and gluconeogenic genes in C57BL/6 and NAS mice fed with a normal chow diet (ND) or a high-fat diet (HFD) for 12 weeks. (**A**) Oxidative genes including *Pparα, Cpt1, Acsl1, Aox1, Acot1*, and *Ucp2* in livers of control (C57BL/6) and NAS mice fed with a ND or a HFD were measured by real-time PCR (n = 5 of each group); (**B**) Lipogenic genes such as *Srebp-1c, Fas, Acc*, and *Pparγ* in livers of control and NAS mice fed with a ND or a HFD were measured by real-time PCR (n = 5); (**C**) Gluconeogenic genes *G6p, Pc, Pepck*, and *aP2* were measured in control and NAS mice fed with a ND or a HFD by real-time PCR (n = 5). Data shown represent the mean ± SEM . Statistically significant differences in gene expression was determined relative to the control mice by a Student’s *t*-test (*p < 0.05; **p < 0.005; ***p < 0.0005).

The improved fatty liver in diet-induced obese NAS mice could be directly attributed to decreased lipogenesis and/or increased oxidation. To differentiate between these possibilities, lipogenic and lipolytic genes were measured by real-time PCR. The expression of peroxisome proliferator-activated receptor alpha (*Pparα*), carnitine palmitoyltransferase 1a (*Cpt1a*), acyl coenzyme A synthetase 1 (*Acsl1*), acyl coenzyme A oxidase 1 (*Aox1*), acyl coenzyme A thioesterase 1 (*Acot1*), and uncoupling protein 2 (*Ucp2*) were significantly (*p* < 0.05) induced in the NAS liver compared with the control (Fig. 3A). In contrast, lipogenic genes including sterol regulatory element-binding protein-1c (*Srebp-1c*), fatty acid synthase (*Fas*), acetyl coenzyme A carboxylase (*Acc*), peroxisome proliferator-activated receptor gamma (*Pparγ*), and fatty acid binding protein 4 (*aP2*) were not differentially expressed between NAS and control mice (Fig. 3B,C). Acc protein levels were also similar between the NAS and control mice (Fig. S4). Previous studies have shown that Notch1 inhibition can impair gluconeogenesis by suppressing glucose 6-phosphatase (*G6p*) expression^14^. Consistent with this finding, we also detected reduced expression of *G6p* in the ND-fed group, but no reduction in phosphoenolpyruvate carboxykinase (*Pepck*) or pyruvate carboxylase (*Pc*) mRNA expression (Fig. 3C). These data suggest the possibility that increased fatty acid oxidation may be responsible for the reduced hepatic lipid accumulation in NAS mice. Thus, our findings highlight potential novel involvement of Notch1 signaling in the regulation of hepatic fatty acid oxidative genes.

### Pharmacological and genetic inhibition of Notch1 signaling increases fatty acid oxidation in HepG2 cells

**Figure 4:**
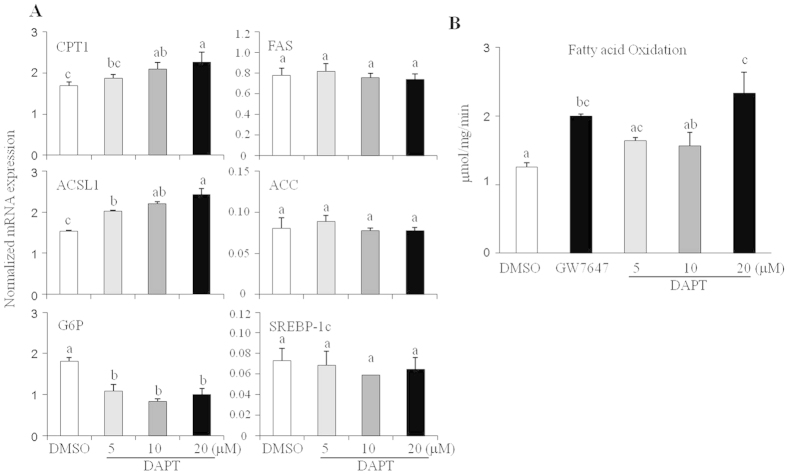
Notch1 inhibition increases the expression of lipid oxidation genes and the rates of fatty acid oxidation in HepG2 cells. (**A**) The Notch signaling inhibitor DAPT increased the expression of oxidative genes but not lipogenic genes. Data shown represent the mean ± SEM, and are representative of three independent experiments. Mean with different letters in each sample indicates a significant difference (*p* < 0.05) by a Student-Newman-Keuls test. (**B**) DAPT increased the rates of fatty acid oxidation. GW7647 (1 μM), a PPARα agonist, was used as a control. Data shown represent the mean ± SD of quadruplicate determinations. Mean with different letters in each sample indicates a significant difference (*p* < 0.05) by a Student-Newman-Keuls test.

The reduced hepatic fatty acid accumulation in NAS mice suggests that Notch1 signaling can act in a cell-autonomous manner. To directly test the hypothesis that Notch1 signaling may affect the expression of genes involved in hepatic fatty acid oxidation, the human liver cancer cells, HepG2, were treated with DAPT. In agreement with our observation in NAS mice, DAPT treatment induced the expression of fatty acid oxidative genes such as *CPT1A* and *ACSL1* in a dose-dependent manner. However, *G6p* expression was suppressed by DAPT, and lipogenic genes (*FAS, ACC, SREBP-1c*) were not differentially expressed (Fig. 4A). To directly test the effect of Notch1 inhibition on fatty acid oxidation, HepG2 cells were treated with DAPT and fatty acid oxidation was measured using [^14^C] palmitate. Comparable to the effects of treatment with the PPARα agonist GW7647, Notch1 inhibition also increased the rate of fatty acid oxidation (Fig. 4B).

**Figure 5:**
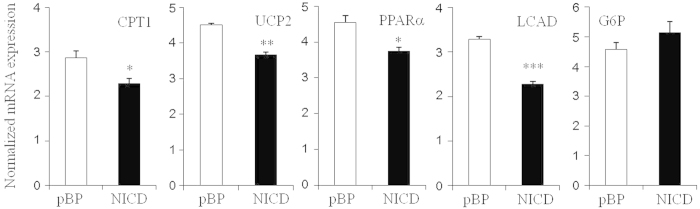
Notch1 activation decreases expression of lipid oxidation genes in HepG2 cells. Effect of Notch1 signaling activation on oxidative genes in HepG2 cells. HepG2 cells were infected with lentivirus expressing NICD1 (NICD1) or control plasmid for 24 hours and gene expression was measured by real time PCR. Data are expressed as the mean ± SEM. Statistically significant differences in gene expression were determined relative to the control by a Student’s *t*-test (*p < 0.05; ***p < 0.0005).

Notch1 receptors are activated upon binding ligands, leading to γ-secretase-dependent generation of NICD1 followed by translocation to the nucleus and transcription of target genes^10^,^13^,^19^. To further verify the regulation of Notch1 on oxidative genes, the activated form of Notch1, NICD1, was transiently expressed in HepG2 cells. Consistent with our data, the activation of Notch1 signaling suppressed the expression of oxidative genes (Fig. 5). Together, these data show cell-autonomous effects of Notch1 signaling in hepatic lipid metabolism.

### Gain and loss of Notch1 function affects lipid accumulation in adipocytes

To further explore the roles of Notch1 signaling in lipid accumulation, epididymal fat tissue was isolated from control and NAS mice, and lipolytic genes were subjected to real-time PCR analysis. The expression levels of *Pparα* and *Cpt1a* were increased in the white fat tissues of NAS mice (Fig. S5). The expression levels of other oxidative genes, *Acsl1* and *Ucp2*, also showed a tendency to be increased in adipose tissues of NAS mice. To investigate the role of Notch1 in lipid accumulation in adipocytes, preadipocyte 3T3-L1 cells and mesenchymal C3H10T1/2 cells were used. To mimic Notch activation, C3H10T1/2 cells were infected with the NICD1-expressing retroviral plasmid pBabe-NICD or empty virus harboring control pBabe-Puro, and expression of oxidative genes was measured. The expression levels of the oxidative genes *Acot1, Acsl1, Ucp2, Aox1*, and *Pparα* were repressed by NICD1 expression in 3T3-L1 cells (Fig. S6). Interestingly, *G6p* expression was not repressed in preadipocytes, suggesting liver-specific regulation of *G6p* expression by Notch signaling. In parallel studies, NICD1 expression in C3H10T1/2 cells also exhibited similar effects on these oxidation genes (Fig. S6).

To examine the influence of NICD1 on lipid accumulation in adipocytes, NICD1-expressing and control cells were differentiated into adipocytes, and lipid accumulation was evaluated. Gain of function studies exhibited enhanced lipid accumulation with increased expression of adipocyte markers in both C3H10T1/2 and 3T3-L1 cells (Fig. S7). The effects of Notch activation were also investigated in fully-differentiated C3H10T1/2 adipocytes. Consistently, forced expression of NICD1 in mature adipocytes also suppressed the expression of oxidative genes (Fig. S8).

**Figure 6:**
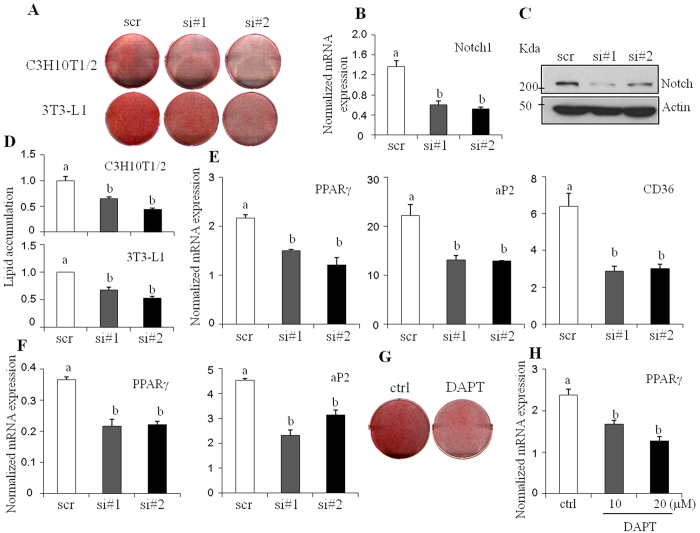
Knockdown of Notch1 by siRNAs decreases lipid accumulation in adipocytes. (**A**) C3H10T1/2 and 3T3-L1 cells were transiently transfected with scrambled (scr) or siRNAs (#1 and #2) targeting Notch1, and induced to differentiate into adipocytes for 7 days. Lipid accumulation was assessed by Oil red O staining. (**B,C**) Two independent siRNAs effectively reduced *Notch1* mRNA (**B**) and protein expression (**C**) in C3H10T1/2 cells. (**D**) Lipid accumulation was quantified by measuring the extracted Oil red O dye at 520 nM. (**E**) Expression of *Pparγ* and its target genes were measured by real-time PCR. (**F**) Fully-differentiated mature C3H10T1/2 adipocytes were transfected with scrambled (scr) or siRNAs (#1 and #2) targeting Notch1 and expression of *Pparγ* and *aP2* were measured by real-time PCR. (**G,H**) Mature C3H10T1/2 adipocytes were treated with DAPT (20 μM) for 48 hours, and lipid accumulation was assessed by Oil red O staining (**G**). (**H**) *Pparγ* expression was measured by real-time PCR. Data are expressed as the mean ± SEM. Mean with different letters in each sample indicates a significant difference (*p* < 0.05) by a Student-Newman-Keuls test.

To test the effect of Notch1 suppression on lipid accumulation, C3H10T1/2 cells expressing scrambled or Notch1 siRNAs were differentiated into adipocytes to induce lipid accumulation. Suppression of Notch1 expression decreased lipid accumulation as assessed by Oil red O staining (Fig. 6A,D). Consistent with previous reports^20^,^21^, cells transfected with two independent siRNAs were compared with scrambled siRNA-transfected cells and shown to successfully reduce *Notch1* mRNA and protein expression (Fig. 6B,C). Furthermore, Notch1 knockdown suppressed the expression of *Pparγ* and its downstream target genes *aP2* and *Cd36* (Fig. 6E). To further examine the roles of *Notch1* signaling in fully differentiated adipocytes, Notch1 was blocked in mature adipocytes and the effects on lipid accumulation were assessed. Notch1 silencing in the differentiated C3H10T1/2 adipocytes suppressed the expression of *Pparγ* and *aP2* (Fig. 6F). Furthermore, DAPT treatment in differentiated adipocytes consistently decreased lipid accumulation and expression of *Pparγ* (Fig. 6G,H). Taken together, these data further corroborate the conserved role of Notch1 in lipid metabolism of hepatocytes and adipocytes.

## Discussion

Hepatic steatosis, the most common form of liver disease, is characterized by high levels of triacylglycerol^22^. The regulatory mechanism of hepatic lipid catabolism is clearly an integral part of hepatic steatosis^23^. Lipid oxidation is controlled by various factors including PPARα and mitochondrial enzymes^24^. In contrast, lipogenesis is catalyzed by SREBP-1c through the activation of FAS or ACC, and by PXR through PPARγ, CPT1, and SCD1^23^,^25^,^26^. Other regulators such as CAR, LXR, and FXR have also been shown to regulate lipogenesis and beta-oxidation^27^. Thus, better understanding of the anabolic and catabolic pathways involved in lipid metabolism may provide another avenue for the intervention of liver disease.

Notch signaling is an evolutionarily conserved pathway that regulates various cell characteristics including cell differentiation and cell fate decision^10^. In the present study, we show that high-fat diet-induced hepatic lipid accumulation was prevented in Notch1-deficient mice. These effects were associated with the induction of oxidative genes without clear effects on the expression of lipogenic genes. Furthermore, the regulation of oxidative genes by Notch1 signaling was consistently observed in adipose tissues. Gain of function reduced the expression of lipolytic genes and lipid accumulation in adipocytes. These results indicate that Notch1 signaling plays a conserved role in lipid metabolism through actions on the expression of oxidative genes.

Pajvani *et al.,* demonstrated that Notch inhibition in combination with *Foxo* deletion can suppress G6P expression, resulting in lower glucose levels^14^. In agreement, *G6P* induction was also detected in the regular chow diet-fed NAS mice. *Pepck, Pc*, and other critical genes in gluconeogenesis were not affected in livers from NAS mice or in *Foxo* and *Notch1* haploinsufficient mice^14^. Thus, it is likely that lowered fasting glucose levels with increased insulin sensitivity may be attributed to the reduced expression of G6P. Whole body deficiency of G6P is characterized by hypoglycemia, hyperlipidemia, and hepatic disorder^28^,^29^. However, mice with liver-specific deletion of G6P exhibit normoglycemia in the fed state^30^. Thus, it seems questionable that G6pc alone in the liver is sufficient to lower glucose levels. Improvement of insulin sensitivity in Notch1-deficient mice could be the combined result of G6P and other effects on metabolism. In agreement with this hypothesis, previous studies have shown that Notch1 signaling can regulate the hepatic lipogenic program through actions on lipogenic gene expression^16^. Our current data reveal a previously unknown role for Notch1 signaling in the regulation of oxidative genes. Reduced levels of G6P and increased expression of lipid oxidation genes may explain the increased insulin sensitivity observed in Notch1-deficient mice.

The present study employed mice ubiquitously expressing Notch1 antisense driven by the mouse mammary tumor virus long terminal repeat promoter. These mice were more insulin sensitive under high-fat diet-fed obese conditions compared with the wild type control mice. Similar to the effects in hepatocytes, the expression levels of oxidative genes in adipocytes were also increased. However, body weight gain, and food intake upon HFD feeding were not different in NAS mice compared with control mice, suggesting that enhanced insulin sensitivity in the NAS mice may be attributed to the actions of increased expression of hepatic oxidative genes. At present, the reason for the differential impacts of Notch1 signaling on lipid accumulation in adipose tissue and liver are unclear. This may be due to the redundant effect of other Notch1 family members in adipose tissues. Indeed, Notch1, 2, 3, and 4 are also expressed in fat tissues, suggesting potential redundant effects of Notch family members in fat tissues. Alternatively, liver-specific or adipose tissue-specific Notch1-deleted mice may exhibit tissue-specific metabolic effects on Notch1 signaling due to pleiotropic effects of Notch1 signaling in various tissues. Indeed, adipose-specific inactivation of Notch1 reduced fat mass and improved insulin sensitivity, corroborating the conserved roles of Notch1 signaling in adipose tissues^24^. Thus, it is reasonable to suggest that Notch1 may play a conserved role in hepatocytes and adipocytes. However, liver-specific Notch1-deleted mice will be required to definitively show hepatocyte-specific effects of Notch1 signaling in fatty liver, as well as metabolic effects, in the future.

Our studies show that the Notch1 signaling pathway can regulate the expression of PPARα and lipid oxidation genes. It remains to be determined how Notch1 signaling regulates the expression of these oxidative genes. One possibility is that Hes1 may function as an inhibitory mediator of Notch signaling. Previous studies have shown that Hes1, a direct target of Notch signaling, behaved as a transcriptional repressor through its binding to the E-Box region on target promoters^25^. Alternatively, NICD1 itself may regulate the expression of lipid oxidation genes indirectly by modulating transcriptional activators or repressors that in turn affect the expression of oxidative genes. Indeed, this is the case in the regulation of *G6p* and *Fas* expression by NICD1^14^,^16^. Determining whether there are direct effects of Hes1 and NICD on promoters of genes that encode lipid oxidation proteins is an intriguing avenue that should be explored in the future.

In the present study, we identified Notch1 signaling as a key determinant of triacylglycerol metabolism in response to a high fat diet, and we further revealed novel biological actions of Notch1 signaling pathways on the regulation of lipid oxidation. Additionally, our data suggest that inhibition of γ-secretase or the Notch signaling pathway could be a novel strategy to prevent and treat fatty liver disease associated with obesity.

## Materials and Methods

### Animal studies

All animal studies were carried out in accordance with, and with approval from, the Animal Research Committee of Sungkyunkwan University. Male C57BL/6 (8-week- old) mice were purchased from Central Lab Animal Inc. (Seoul, Korea) and housed in controlled temperature (24 ± 2 °C) and humidity (50 ± 10%) conditions under a 12-hour light and 12-hour dark cycle. Notch1 antisense transgenic (NAS) mice were generated as described previously^12^. These mice were individually housed and allowed free access to water and food (Solid feed 5L79, Orient. Co. LTD, Seoul, Korea). After an adaptation period of 1 week, 9-week-old mice were randomly assigned into four groups. One group of each C57BL/6 and NAS mice were fed ND (normal chow diet), while the second group of C57BL/6 and NAS mice were given a high-fat diet containing 60% fat (HFD; Research Diets Inc., NJ, USA) for 12 weeks. Body weight was measured twice per week, and food intake was determined three times per week.

For glucose tolerance tests, mice were fasted for 16 hours. Their glucose levels from tail-vein blood were determined prior to, and 30, 60, 90, and 120 minutes after i.p. injection of glucose (2 g/kg). For insulin tolerance tests, random fed-mice were injected i.p. with insulin (Humulin R, Eli Lilly, 1 U/kg). Glucose levels were determined prior to, and 30, 60, 120, 180, and 210 minutes after injection, using an Accu-Chek Active glucometer (Roche, Seoul, Korea). At the end of the experiments, mice were fasted for 16 hours and anesthetized by inhalation of ether followed by cardiac puncture. For histological studies, harvested tissues were fixed in 10% formaldehyde for 2 days and embedded in paraffin. Paraffin blocks were sectioned at a thickness of 5 μm and subjected to hematoxylin and eosin staining.

### Cell culture

HepG2, C3H10T1/2, and 3T3-L1 cells were purchased from the American Type Culture Collection (Manassas, VA, USA). HepG2 cells were maintained in DMEM media supplemented with 10% fetal bovine serum (FBS) and 1% Pen/Strep. C3H10T1/2 and 3T3-L1 cells were maintained and differentiated into adipocytes as previously described^31^. For differentiation, confluent cells were induced in DMEM supplemented with 10% FBS, 1 μM dexamethasone (Sigma, St. Louis, MO), 0.5 mM isobutyl-1-methylxanthine (Sigma), and 5 μg/ml insulin (Sigma) for 2 days. Every 2 days, cells were refreshed with media containing FBS and insulin. After 6–8 days of differentiation, cells were fixed with 4% paraformaldehyde in PBS at room temperature for 4 hours, and stained with 0.5% Oil Red O (ORO, Sigma). ORO stained cells from at least two independent experiments were quantified by extracting dye with isopropanol. Absorbance was measured at 520 nm. Retroviral or lentivrial overexpression of NICD was performed using pBabe-puro and the packaging cell line of the Phoenix system as described previously^31^.

### Protein expression

For Western blotting, cells were harvested in cell lysis buffer (1% Triton X-100 and 0.5% sodium deoxylcholate) with protease inhibitor cocktail tablets (11 836 153 001, Roche, Mannheim, Germany). Total cell lysates were separated by 10% sodium dodecyl sulfate-polyacrylamide gel electrophoresis (SDS-PAGE) and transferred to Amersham Hybond ECL nitrocellulose membranes (RPN 303D; GE Healthcare, Piscataway, NJ, USA) as described previously^32^. Anti-Notch1 (Sc 6014R) was obtained from Santa Cruz Biotechnology (Santa Cruz, CA, USA). Anti-IRS and pIRS1 (pTyr^612^) were purchased from Sigma-Aldrich (St. Louis, MO). Anti-AKT and pAKT(Ser473) were purchased from Cell Signaling (Beverly, MA). Anti-β-actin (A5316) antibody was purchased from Santa Cruz Biotechnology. Visualized protein bands were quantified by densitometry using the Image J software (National Institute of Health, Bethesda, MD).

### Quantitative real-time PCR

To isolate total RNA from mouse tissues, homogenized TRIzol samples were centrifuged and total RNAs were isolated. Isolated RNA was further cleaned by phenol-chloroform extraction, followed by ethanol precipitation. Total RNA (500 ng) and 300 pmol random primers were mixed and heated at 65 °C for 5 minutes, and reverse transcribed using RTase M-MLV buffer, dNTP mixture, and RTase M-MLV (2640A, Takara, Ohtsu, Japan) at 37 °C for 50 minutes. The cDNA was amplified by Thermal cycler dice (Takara) using Power SYBR Premix Ex Taq (RP041A, Takara) and specific primer sets as previously described and shown below^31^. Expression was normalized to 36B4. All real-time PCRs were performed at least twice. △cycle threshold (CT) was used to calculate the differences between the target CT value and the control (36B4) for each sample: △CT = CT_target_ − CT_control_. The relative expression level was calculated using 2^−△CT^. The oligonucleotide primer (Integrated DNA Technologies, San Diego, CA) sequences used for PCR were as follows: *Pparα* F, 5′-atgccagtactgccgttttc-3′ and *Pparα* R, 5′- ccgaatctttcaggtcgtgt-3′; *Aox1* F, 5′- caccattgccattcgataca-3′ and *Aox1* R, 5′- tgcgtctgaaaatccaaaatc-3′; *Acot1* F, 5′-gatcgcctcaaggatgttgt-3′ and *Acot1* R, 5′- atgatctggggcttctcctt-3′; *Cpt1a* F, 5′-gatgtggacctgcattcctt-3′ and *Cpt1a* R, 5′-tcttgtaatgtgcgagctg-3′; *Ucp2* F, 5′-acagccttctgcactcctg-3′ and *Ucp2* R, 5′- ggctgggagacgaaacact-3′; *Acsl1* F, 5′-accatgtacgatggcttcca-3′ and *Acsl1* R, 5′- tcatagggctggtttggctt-3′; *Fas* F, 5′-gctgctgttggaagtcagc-3′ and *Fas* R, 5′- agtgttcgttcctcggagtg-3′; *Acc* F, 5′-gcagccctgggcacag-3′ and *Acc* R, 5′-gggaatacccgtgggagtagtt-3′; *G6p* F, 5′-tctgtcccggatctaccttg-3′ and *G6p* R, 5′-gaaagtttcagccacagcaa-3′; *Pc* F, 5′- cggcagggcggagctaacat-3′ and *Pc* R, 5′-tttggggaggcaacaggggc-3′; *Pepck* F, 5′-atcatctttggtggccgtag-3′ and *Pepck* R, 5′-catggctgctcctacaaaca-3′; *Notch1* F, 5′-ctggaccccatggacatc-3′ and *Notch1* R, 5′- aggatgactgcacacattgc-3′; *Pparγ* F, 5′-ccattctggcccaccaac-3′ and *Pparγ* R, 5′- aatgcgagtggtcttccatca-3′; *aP2* F, 5′-caccgcagacgacaggaag-3′ *aP2* R, 5′-gcacctgcaccagggc-3; Human *PPARα* F, 5′-gcactggaactggatgacag-3′ and human *PPAR*α R, 5′-tttagaaggccaggacgatct-3′; human *FAS* F, 5′-caggcacacacgatggac-3′ and human *FAS* R, 5′-cggagtgaatctgggttgat-3′; human *ACC* F, 5′-gctggtccacatgaacagg-3′ and human *ACC* R, 5′-gccttctggatattcaggacttt-3′; human *SREBP-1c* F, 5′-cgctcctccatcaatgaca -3′ and human *SREBP-1c* R, 5′- tgcgcaagacagcagattta-3′; human *CPT1A* F, 5′-caatcggactctggaaacg-3′ and human *CPT1A* R, 5′-ccgctgaccacgttcttc-3′; human *UCP2* F, 5′-tgaaagccaacctcatgaca-3′ and human *UCP2* R, 5′-gatgacagtggtgcagaagc-3′; human *ACSL1* F, 5′-gcttttgtgaaagcaacagaga-3′ and human *ACSL1* R, 5′-ggcgagaggcaagaaagata-3′; human *LCAD* F, 5′-ggatctgtactccgcagctatt-3′ and *LCAD* R, 5′-ctaaaacctgggcctgaaca-3′; human *G6P* F, 5′-ccctgtaacctgtgagactgg-3′ and *G6P* R, 5′-aaagagtagatgtgaccatcacgta-3′;

### siRNA knockdown of Notch1

siRNA knockdown of Notch1 was carried out as described previously^20^. Two independent small interfering RNA (siRNA) sequences targeting Notch1 and a nonspecific negative control were purchased from Genolution Pharmaceutical Inc. (Seoul, Korea). *Notch1* specific siRNA#1 was sense 5′- CCAACAAGGACAUGCAGAACAACAA UU-3′. Notch1 specific siRNA#2 was sense 5′-UGAGACAGGCAACAGUGAAGAAGAA UU-3′. The non-specific sequence for the negative control was sense 5′-CCUCGUGCCGUUCCA UCAGGUAGUU-3′. Cells were transfected with 20 pmol siRNA using Lipofectamine RNAiMAX (Invitrogen 13778-075) for 16 hours. After 48 hours, total RNA was extracted to measure the expression of oxidation genes by real-time PCR. To assess the effects on lipid accumulation, the transfected cells were induced and differentiated into adipocytes for 7 days followed by ORO staining or total RNA extraction for real-time PCR. Transfections were carried out in triplicates.

### Fatty acid oxidation

HepG2 cells were seeded in 24-well plates at a density of 2.5 × 10^5^/well for 24 hours. Subsequently, the cells were loaded with free fatty acids (100 μM palmitic acid and 100 μM oleic acid) containing 0.5% bovine serum albumin (BSA, Bovogen Biologicals, Melbourne, Australia) for 24 hours. The following day, cells were treated with DAPT (5, 10, or 20 μM) or 1 μM GW7647 in DMEM containing 0.45 mM [^14^C] palmitate (57 mCi/mM; Perkin Elmer) conjugated to 1 mg/ml FA-free BSA for a further 24 hours. The medium was removed to a sealed container and the ^l4^CO_2_ was extracted by mixing with 0.5 ml 1 M NaOH and 0.5 ml concentrated HCl. The amount of ^14^CO_2_ was measured using a liquid scintillation counter. The total protein concentrations of cells were determined for normalization.

### Statistical analysis

Data are presented as the mean ± SEM. Differences in gene expression and lipid accumulation were analyzed using a two-tailed unpaired Student’s *t*–test or analysis of variance (ANOVA) followed by a Student-Newman-Keuls test. All computations were performed using statistical analysis software (PASW Statistics 17). Statistical significance was considered when a *p* value was less than 0.05.

## Additional Information

**How to cite this article**: Song, N.-J. *et al.* Notch1 deficiency decreases hepatic lipid accumulation by induction of fatty acid oxidation. *Sci. Rep.* **6**, 19377; doi: 10.1038/srep19377 (2016).

## Supplementary Material

Supplementary Information
