# Supplementary material for: Notch1 deficiency decreases hepatic lipid accumulation by induction of fatty acid oxidation
Source: Sci Rep. 2016 Jan 20;6:19377. doi: 10.1038/srep19377 (PMC4726366; doi:10.1038/srep19377)

Supplementary Information

Notch1 deficiency decreases hepatic lipid accumulation by induction of fatty acid oxidation

No-Joon Song[1,#], Ui Jeong Yun[2,#], Sunghee Yang[2], Chunyan Wu [3], Cho-Rong Seo[1], A-Ryeong Gwon[2,4], Sang-Ha Baik[2], Yuri Choi[2], Bo Youn Choi[2], Bahn Gahee[2], Suji Kim[1], So-Mi Kwon[1], Jin Su Park[2], Seung Hyun Baek[2], Tae Joo Park[5], Keejung Yoon[6], Byung-Joon Kim[4], Mark P. Mattson[7], Sung-Joon Lee[3], Dong-Gyu Jo[2,*], Kye Won Park[1,*]

[1]Department of Food Science and Biotechnology, Sungkyunkwan University, Korea. [2]School of Pharmacy, Sungkyunkwan University, Korea. [3]Department of Biotechnology, Graduate School of Life Sciences & Biotechnology, BK21-PLUS program, Korea University, 136-713 Seoul Korea. [4]Department of Internal Medicine, Graduate School of Medicine, Gachon University of Medicine and Science, [5]School of Nano-Bioscience and Chemical Engineering, Ulsan National Institute of Science and Technology, [6]Department of Genetic Engineering, Sungkyunkwan University, Korea. [7]Laboratory of Neurosciences, National Institute on Aging Intramural Research Program, Baltimore, Maryland, USA

[#] These authors contributed equally.

[*]Corresponding authors: Dong-Gyu Jo, PhD. School of Pharmacy, Sungkyunkwan University, Suwon 440-746, Korea. Phone: (+82)-31-290-7782 , jodg@skku.edu; Kye Won Park, PhD. Department of Food Science and Biotechnology Sungkyunkwan University, Suwon 440-746, Korea. Phone: (+82)-31-290-7804 , kwpark@skku.edu

Supplementary Information:

Figure S1
Figure S2
Figure S3
Figure S4
Figure S5
Figure S6
Figure S7
Figure S8
Original blots

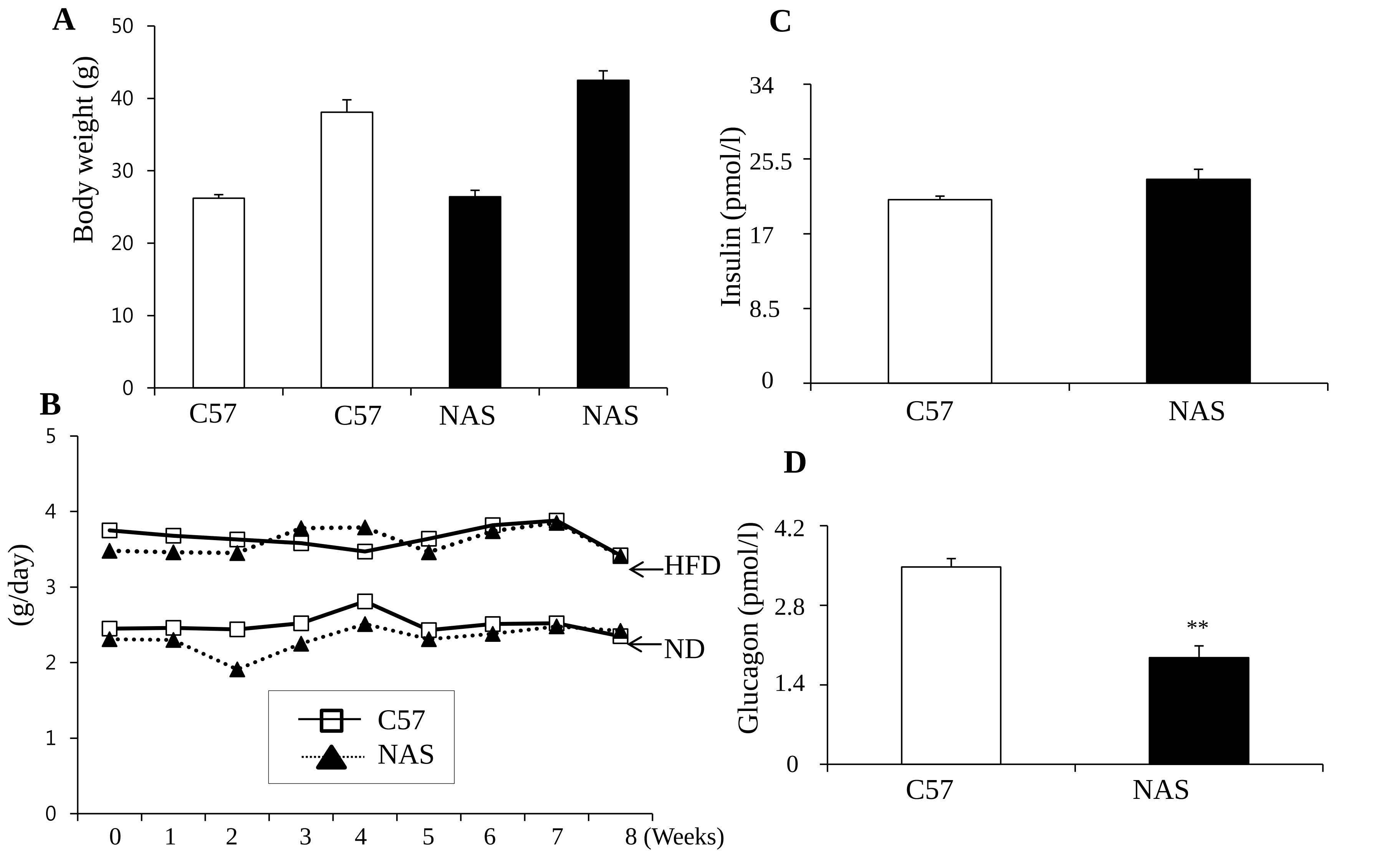

Figure S1. Body weight, food intake, insulin, and glucan levels in Notch1 insufficient mice. (A-C) Body weight (A), food intake (B), insulin (C), levels were not different in high fat diet fed (HFD) C57BL/6 mice (C57) and Notch1 antisense transgenic (NAS) mice. (D) Plasma glucagon level was decreased in NAS mice (n=5 of each group). Statistically significant differences in the control normal chow fed C57BL/6 (C57-ND) and Notch1 antisense transgenic (NAS) mice or high fat diet fed C57BL/6 mice (C57-HFD) and high fat diet fed Notch1 antisense transgenic mice (NAS-HFD) were determined using Student's t-test (** P < 0.005).

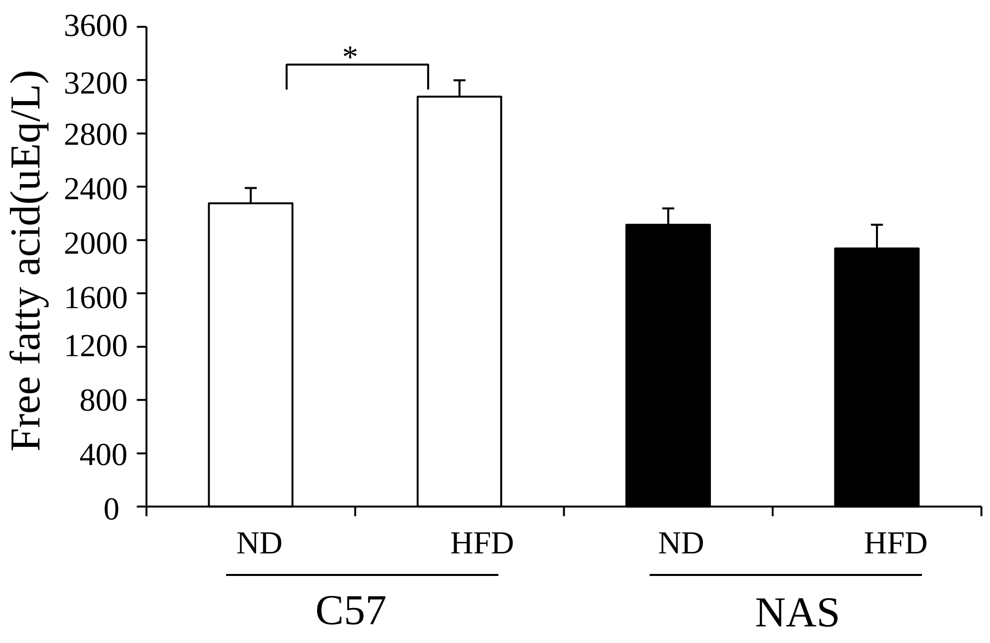

Figure S2. Serum free fatty acid levels from normal diet (ND) or high fat diet fed (HFD) C57BL/6 mice (C57) and Notch1 antisense transgenic (NAS) mice were determined. Statistically significant differences in the control normal chow fed C57BL/6 (C57-ND) and Notch1 antisense transgenic (NAS) mice or high fat diet fed C57BL/6 mice (C57-HFD) and high fat diet fed Notch1 antisense transgenic mice (NAS-HFD) were determined using Student’s t-test (* $P < 0.05$).

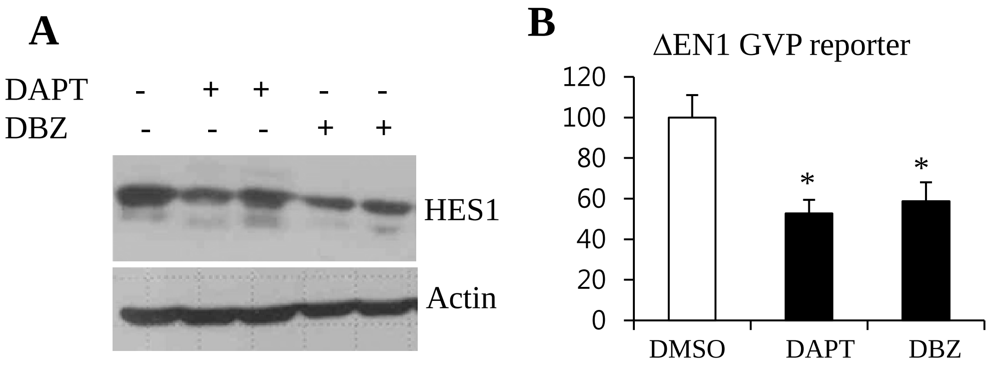

Figure S3. Notch inhibitors decrease Notch activation. (A) Western blot analysis of HES1 from DAPT and DBZ treated HepG2 cells. HepG2 cells were treated with Notch signaling inhibitors DAPT or DBZ (5 μM) and expression of HES1 was measured by western blot analysis. (B) HepG2 cells transfected with the CSL luciferase reporter (notch-specific reporter) and an expression vector coding for ΔEN (γ-secretase cleavage sites fused to GAL4-VP16) were treated with DAPT or DBZ (5 μM) and luciferase activity was measured. Data are expressed as the means ± SEM. Statistically significant differences in gene expression was determined relative to the control by the Student's *t*-test (* $P < 0.05$).

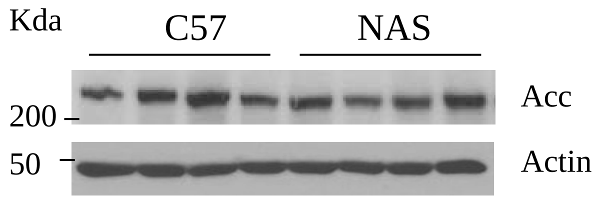

Figure S4. Western blot analysis of Acc protein in livers from control (C57) and Notch1 antisense transgenic (NAS) mice. Hepatic Acc protein levels were not different in high fat diet fed control and NAS mice.

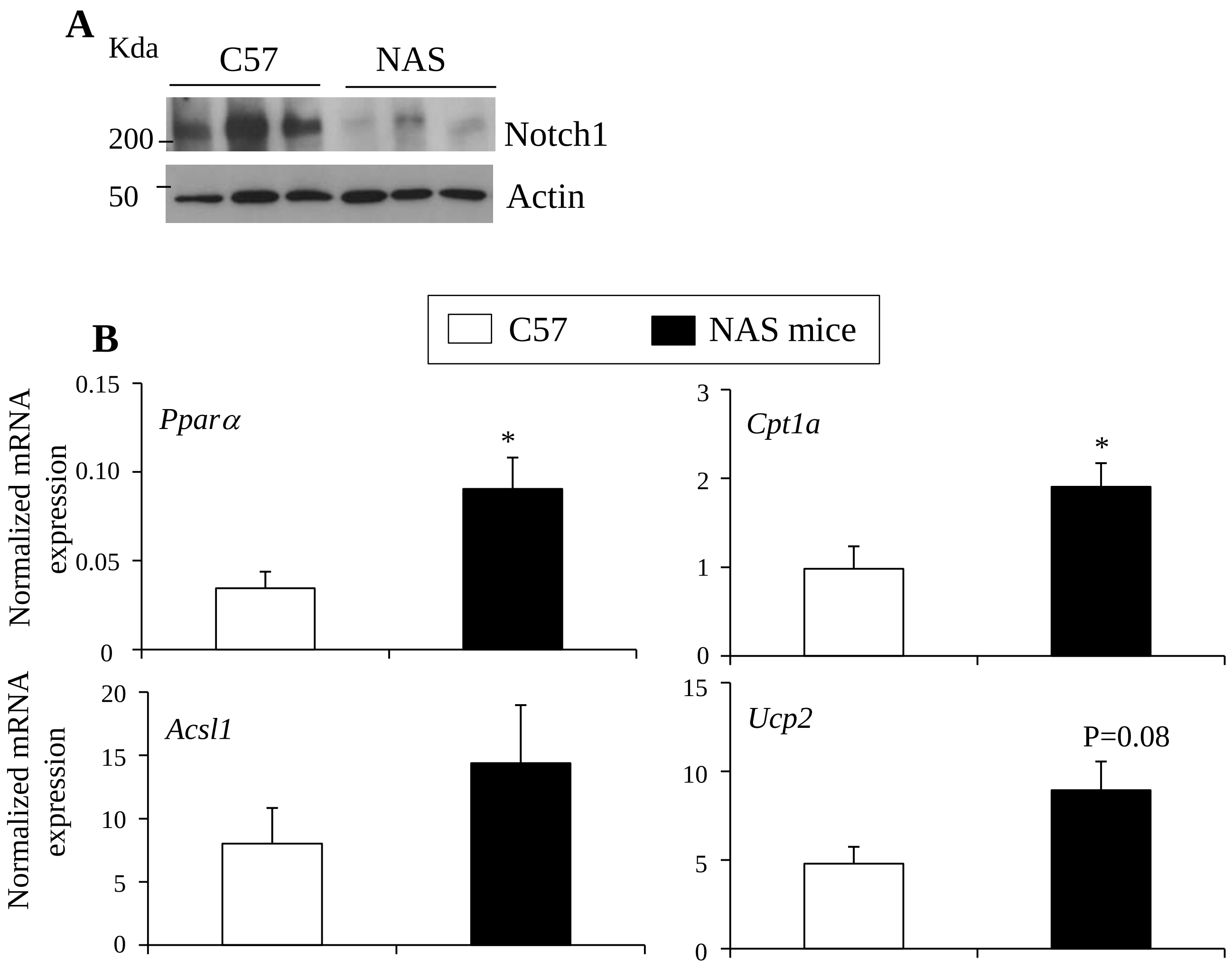

Figure S5. Notch1 deficient mice induce expression of oxidative genes in epididymal white adipose tissues. (A) Reduced Notch1 expression in epididymal white adipose tissues from high fat diet fed C57BL/6 mice (C57) and Notch1 antisense transgenic (NAS) mice. Notch1 protein expression was measured by immunoblotting. (B) Oxidative genes including *Pparα*, *Acsl1*, *Cpt1a*, and *Ucp2* in epididymal fats of HFD control (C57BL/6) and NAS mice were measured by real time PCR (n=5 of each group). Data shown represent the mean ± SEM. Statistically significant differences in gene expression was determined relative to the control (C57BL/6) mice by the Student's *t*-test (* $P < 0.05$).

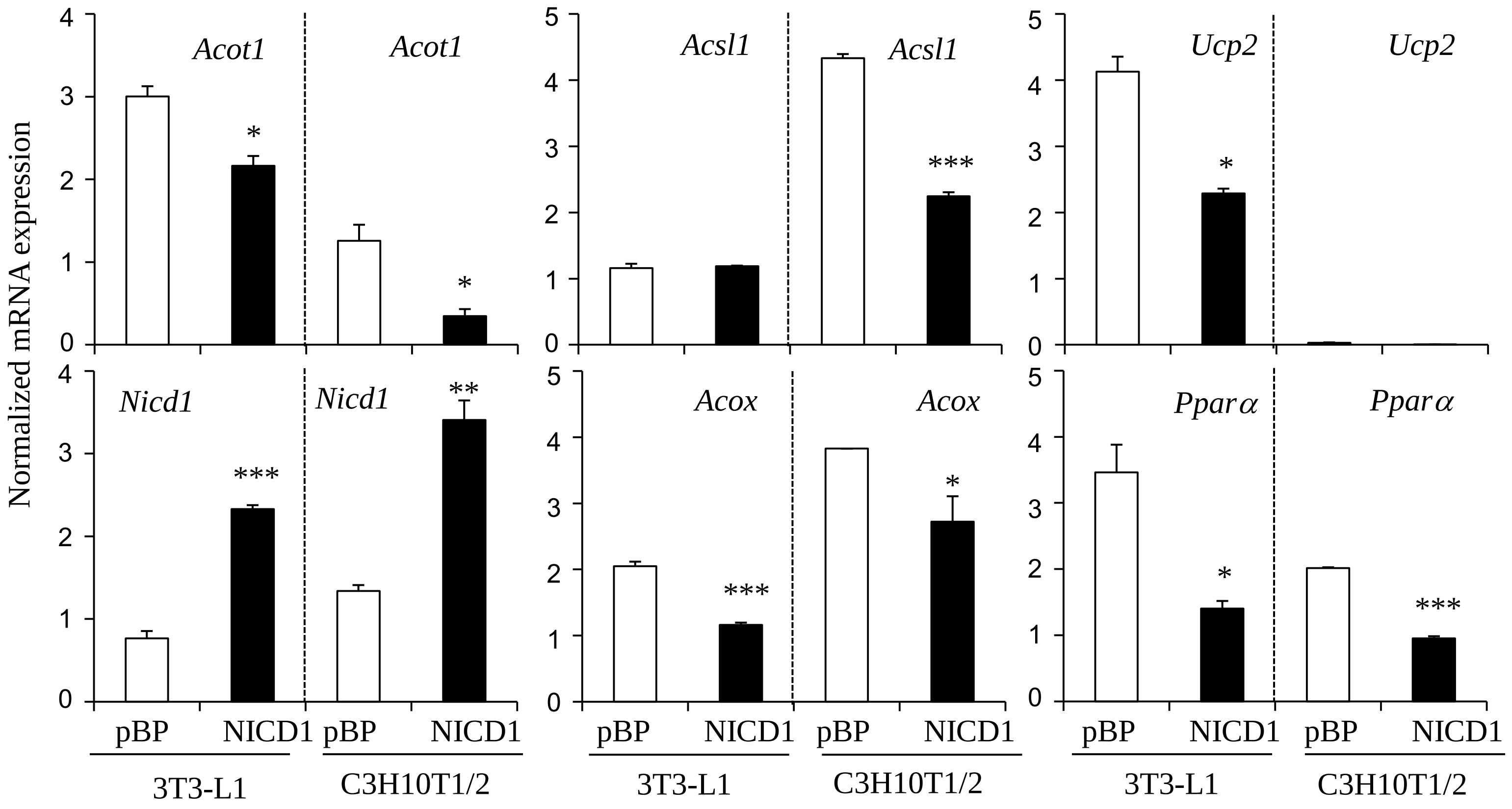

Figure S6. Notch1 gain of function suppressed the expression of oxidative genes in preadipocytes.
3T3-L1 and C3H10T1/2 preadipocytes were infected with retrovirus expressing NICD1 (pBP-NICD1) or virus harboring pBabe-Puro (pBP) empty vector and stable cells were selected with puromycin (2 µg/ml) for 2 weeks. Gene expression in stable preadipocytes was measured by real time PCR. Data were expressed as mean ± SEM. Statistically significant differences in gene expression was determined relative to the control by the Student's *t*-test (*$p < 0.05$; ** $p < 0.005$; *** $p < 0.0005$).

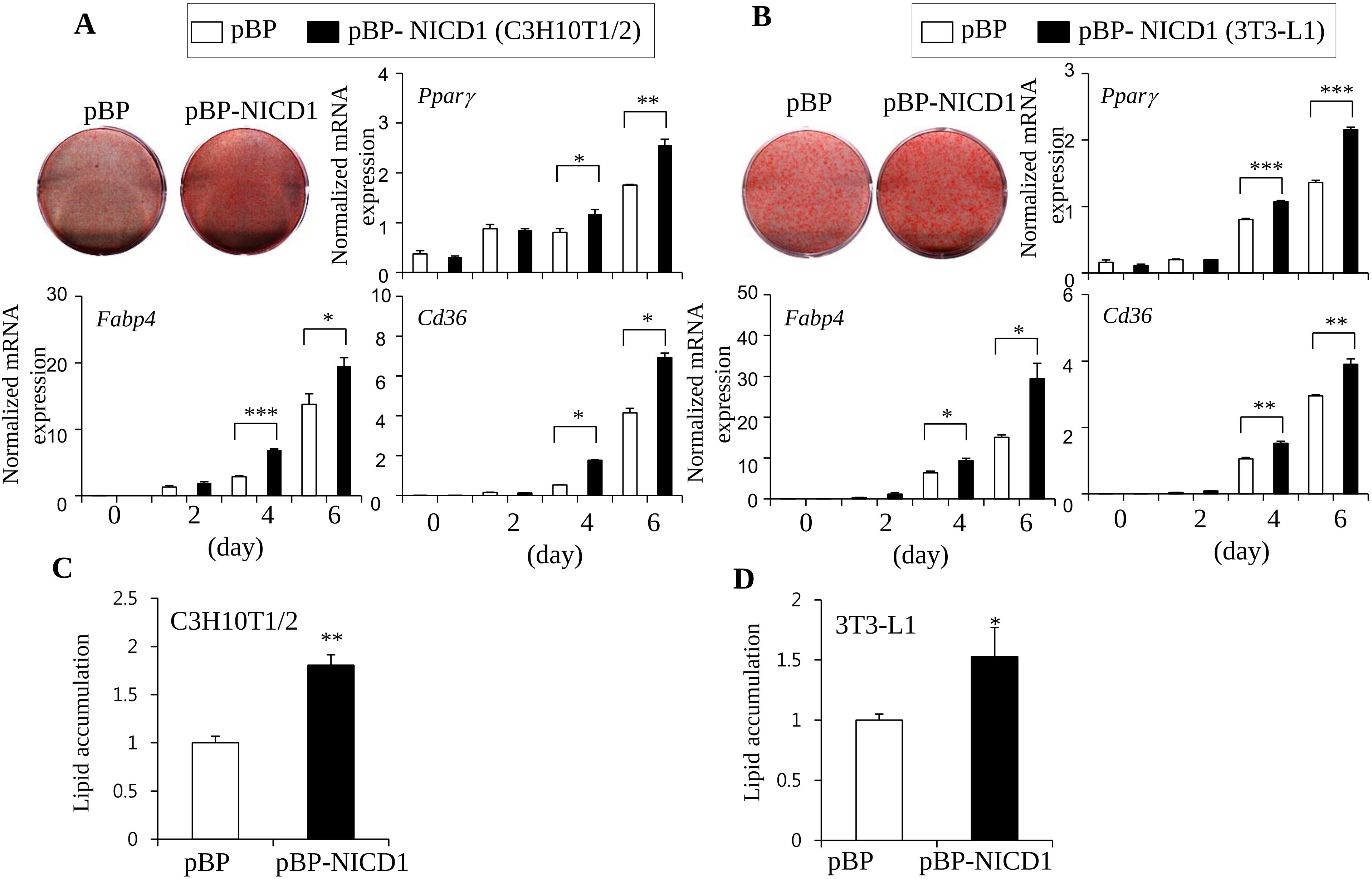

Song et al., Fig S7

Figure S7. Notch1 gain of function increases lipid accumulation and induces expression of adipocyte markers during adipocyte differentiation of C3H10T1/2 and 3T3-L1 cells. (A) C3H10T1/2 cells were infected pBabe-puro (pBP) or pBabe-NICD1 harboring retrovirus (pBP-NICD1) and stable cells were selected with puromycin (2 μg/ml) for 2 week. Stable cells were induced into adipocytes and lipid accumulation on day 6 was assessed by Oil red O staining. (B) 3T3-L1 cells were infected with pBabe-puro (pBP) or pBabe-NICD1 harboring retrovirus (pBP-NICD1) and stable cells were selected with puromycin (2 μg/ml) for 1 week followed by adipocyte differentiation for 6 days. Stable cells were induced into adipocytes and lipid accumulation on day 6 was assessed by Oil red O staining. Gene expression was measured by real time PCR. **(**C,D**)** Stable cells were induced into adipocytes and lipid accumulation on day 6 was assessed by Oil red O staining followed quantification. Lipid accumulation was quantified by measuring the extracted Oil red O dye at 520 nM. Data are expressed as the means ± SEM. Statistically significant differences in gene expression was determined relative to the control by the Student's *t*-test (* $P < 0.05$; ** $P < 0.005$; *** $P < 0.0005$).

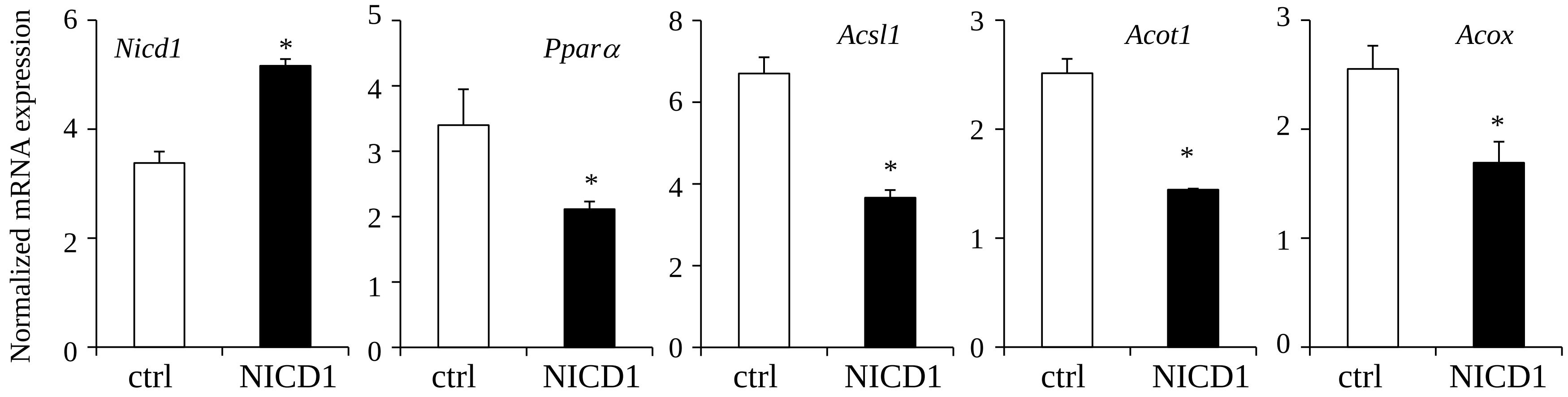

Figure S8. Notch1 gain of function suppressed the expression of oxidative genes in mature adipocytes. C3H10T1/2 preadipocytes were differentiated into mature adipocytes for 8 days and infected with lentivirus expressing NICD1 (NICD1) or control plasmid. Gene expression in NICD 1 and control cells was measured by real time PCR. Data were expressed as mean ± SEM. Statistically significant differences in gene expression was determined relative to the control by the Student's *t*-test (*$p < 0.05$).

Original blots

Fig. 1B

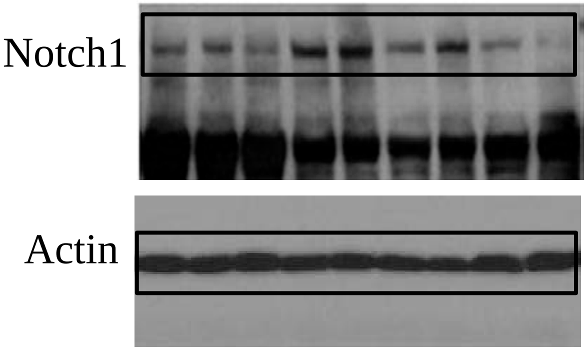

Fig2 A

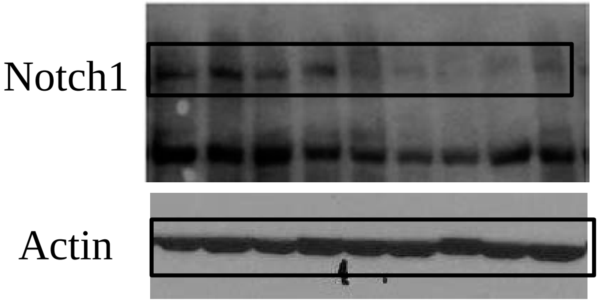

Fig. 2F

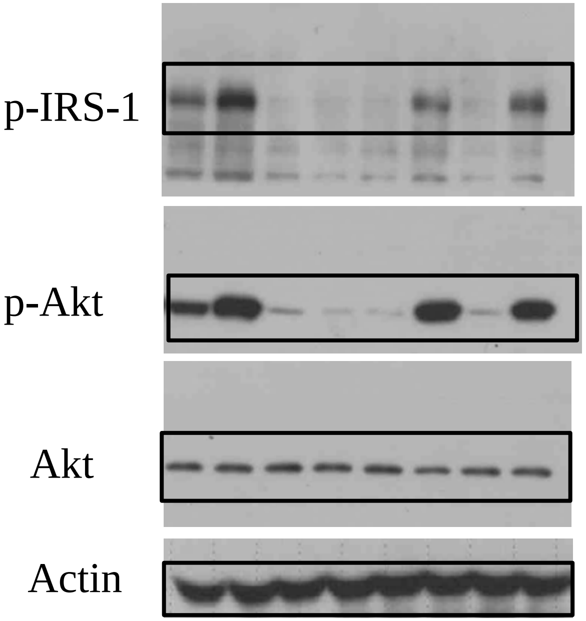

Fig. 6C

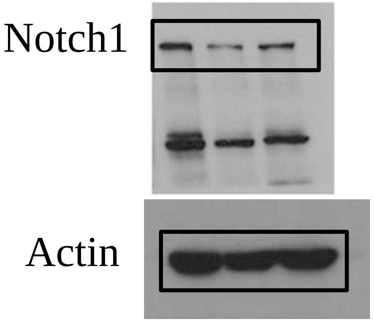

Supplement: Supplementary Information [file srep19377-s1.pdf]
